# Musculoskeletal Disorders and Associated Factors among Vehicle Repair Workers in Hawassa City, Southern Ethiopia

**DOI:** 10.1155/2020/9472357

**Published:** 2020-05-07

**Authors:** Aiggan Tamene, Hailemichael Mulugeta, Tesfaye Ashenafi, Steven M. Thygerson

**Affiliations:** ^1^Department of Public Health, College of Medicine and Health Sciences, Wachemo University, Hosaena, Ethiopia; ^2^Department of Public Health, College of Health Science, Debre Berhan University, Debre Birhan, Ethiopia; ^3^Department of Environmental Health, College of Medical and Health Science, Hawassa University, Awasa, Ethiopia; ^4^Department of Public Health, College of Life Sciences, Brigham Young University, Provo, USA

## Abstract

**Background:**

Vehicle repair work is one of the highest risk professions for work-related musculoskeletal disorders. Globally, only a few published studies have examined the prevalence and determinants of work-related musculoskeletal disorders among vehicle repair workers. Related studies in Ethiopia are even fewer. This study aimed to determine the prevalence of self-reported work-related musculoskeletal disorders and associated factors among vehicle repair workers in Hawassa city, South Ethiopia, 2019.

**Methods:**

An institution-based cross-sectional study was conducted among 344 vehicle repair workers in the Hawassa city. The Nordic Musculoskeletal Questionnaire-Extended (NMQ-E) was used to assess work-related musculoskeletal disorders on nine body regions. Descriptive statistics and multivariable analyses were used to characterize the data and identify factors associated with work-related musculoskeletal disorders.

**Result:**

The twelve-month prevalence of work-related musculoskeletal disorders among this working group was 47.7% with 95% CI (42.7–53.2). Jobs continuously requiring repetitive motions (AOR: 4.29, 95% CI (1.78–10.2)), not having professional training (AOR: 2.04, 95% CI (1.09–3.81)), force exertion when using tools (AOR: 2.40, 95% CI (1.24–4.62)), job stress (AOR:4.54, 95% CI (2.44–8.46)), and regularly lifting, pushing, and pulling loads greater than 20 kg (AOR:4.85, 95% CI (2.65–8.87)) were identified as associated factors.

**Conclusion:**

This study showed a 47.7% prevalence of work-related musculoskeletal disorders. Force exertion, repetitive tasks, manual handling of heavy loads, stress, and lack of training were the identified factors. Ergonomic awareness among workers should be increased through training. In addition, owners should investigate methods to reduce or eliminate risk factors leading to musculoskeletal disorders found among these workers. Automation of high-risk tasks should also be investigated.

## 1. Introduction

Musculoskeletal disorders (MSDs) are injuries or pain that affect the body's musculoskeletal system. These include bones, nerves, tendons, ligaments, joints, cartilage, blood vessels, and spinal discs [1]. However, work-related musculoskeletal disorders (WMSDs) are musculoskeletal disorders that are caused or aggravated primarily by work and by the effects of the immediate environment in which work is carried out [2].

WMSDs are one of the leading causes of activity-limiting diseases among working populations [3]. They have a significant socioeconomic impact and affect the quality of life of those afflicted. These disorders drive up costs for workers, companies, and society in general [4]. The International Labor Organization (ILO) estimates that some 2 million women and men around the world fall victim to work-related diseases every year; this corresponds to over 5,480 deaths every single day. Globally, WMSDs are frequently implicated as one of the leading causes of worker complaints [5]. These disorders are the most frequent health complaint by workers of developed nations [3]. In the developing world, among other things, inadequacy in the workers' understanding of occupational hazards, lack of control measures regarding occupational hazards, and poorly designed workstations are likely to greatly increase the burden of these disorders among workers [6].

Workers involved in occupations such as health care, driving, production/manufacturing, general labor, maintenance, repairing, and cleaning are at the highest risk for MSD [7]. In these occupations, repetitive tasks and tasks requiring body positions to be in awkward positions for long periods of time increase the risk of WMSDs [8]. Epidemiological studies on WMSDs studying several industries reported various WMSD prevalence rates among workers. These studies reported the following prevalence rates: 79% in the manufacturing industry in Brazil [9], 41.5% among Iranian petrochemical industries [10], and 97.3% among textile industry workers in India [11].

WMSD prevalence rates show that vehicle repair work is among the highest risk professions [6, 12]. Studies demonstrate a prevalence of 85% and 58% in India [13, 14], 92% in Malaysia [15], and 77% in Bangladesh [16].

In many of these WMSD studies, a variety of risk factors have been identified and the cause is multifactorial in origin. In particular, individual, psychosocial, ergonomic, and work environment factors have been identified as contributing agents to the worsening of musculoskeletal disorders [6, 12–15].

The global automotive repair and maintenance service industry is expected to grow by double digits during 2018–2022. Africa is projected to be one of the largest markets in terms of growth in this period [17]. According to the urban employment and unemployment survey carried out in Ethiopia, there were 30,538 automotive electronic servicing professionals, 53,612 mechanical repair professionals, 1,613 painting professionals, and 2,348 automotive servicing managers in Ethiopia. Altogether, a total of 88,111 vehicle repair workers were reported to be engaged in this service sector in urban areas [18].

However, despite the presence of such a fairly large number of vehicle repair workforce in Ethiopia, information on WMSDs among these workers is still limited. This study was, therefore, designed with the main objective of assessing the prevalence of WMSDs and secondary objective of identifying factors associated with WMSDs among vehicle repair workers in Hawassa city, Ethiopia.

## 2. Materials and Methods

The study was conducted in the Hawassa city from January 25 to February 22, 2019. Hawassa is the capital of the Southern Nations, Nationalities, and Peoples' Region in Ethiopia. Located on the shores of Lake Hawassa in the Great Rift Valley, the city administration is divided into 8 subcities. The 2015 Population and Housing Census Report estimates that the population of Hawassa is 351,469, making it the third largest city in Ethiopia by inhabitants.

Both government and private-owned vehicle repair workshops are currently operating in the Hawassa city. The most common type of repair workshop in the city is the private garages. The other group of repair workshops is government-owned establishments. These workshops provide services to vehicles owned by governmental offices. According to the information obtained from the city transport bureau, there were a total of 38 private and 3 governmental autorepair workshops registered in the Hawassa city.

The study population was all vehicle repair personnel working in registered garages in Hawassa city, Ethiopia. Workers with a minimum service of 12 months were included. However, workers with congenital insensitivities like scoliosis, with any diagnosis of neurologic symptoms before starting this work, and workers who had joint disease, gout, and trauma during one year before the data collection were excluded from the study.

The sample sizes for objective one and two were calculated separately using a formula for single population for the first objective and double population for the second objective considering different assumptions. Since no studies were found on WMSDs among vehicle repair workers in our context, any attempt to be made towards taking the baseline prevalence from another setting may affect the representativeness of the present study. Thus, in order to increase precision of the result of the study, the maximum sample size assumption with prevalence among workers at *P*=50%, margin of error (d) of 5%, and a 95% level of confidence were considered.

Based on these assumptions, the Epi Info™7 version program yielded a total sample size of 384.

However, since sampling was done from a finite population, the following correction formula was applied:

(1) $$\begin{array}{c} \text{nf}=\frac{\text{ni}}{1+\left(\text{ni}/N\right)}, \end{array}$$

where ni is the sample size calculated from infinite population, nf is the total sample size to be studied, and *N* is the source population (368 workers).

The final sample size for objective one was 188 vehicle repair workers. Considering a 10% nonresponse rate, the final sample size was 207.

The sample size for the second objective was obtained by using a double population formula using factors that had strong associations with the outcome of interest in other literatures [15]. Finally, the largest sample size obtained was the one calculated for the second objective (*n* = 306) and thus used as a final sample size of the study.

An on-site census was conducted to determine the eligibility of the study participants from all the registered automotive vehicle repair workshops in the Hawassa city. A total of 368 workers were recognized as operating in 41 vehicle repair workshops in the city. From these, 297 vehicle repair workers were employed in the private garages and 71 workers were employed in government-owned garages.

Based on the eligibility criteria set, four workers from government-owned facilities and 17 from private autorepair establishments were excluded. This means 347 vehicle repair workers were available for selection. All 347 vehicle repair workers were included in the study to maximize the study precision or accuracy. It was also noted that data quality, feasibility, and available resources would not be compromised since 347 was not an excessively large sample.

Face-to-face interview of participants was employed for data collection using a pretested, well-structured and close-ended questionnaire. WMSD symptoms were measured using the extended version of the standardized Nordic Musculoskeletal Questionnaire (NMQ-E) [19]. An anatomical diagram of nine body regions (neck, shoulder, upper and lower back, hands/wrists, arms, knee, thighs, and feet) facilitated respondents to precisely identify the presence of musculoskeletal symptoms for the preceding 12 months, past one month, and last seven days.

Another questionnaire adapted from prior MSD studies was used in the present study to assess the sociodemographic and personal status of the respondents [20, 21]. The Generic Job Satisfaction scale [22] and the work place stress scale [23] were also used to measure the level of satisfaction and stress associated with the participants.

Anthropometric measurements such as weight in kilogram (kg), height in centimeter (cm), and body mass index (BMI) in kilogram per meter square (kg/m^2^) of the participants were also measured. A digital weight scale was used for assessing weight, and height was measured using a standard meter.

In the present study, self-reported WMSD was considered as the dependent variable. The independent variables were sociodemographic factors (age, sex, educational background, work experience, and monthly income); personal factors (BMI, physical activity, smoking, drinking, khat chewing habits, professional training, and systemic illness history); work environment and ergonomic factors (work load, work posture, work space, repetitive movement, assistive equipment availability, force exertion, ergonomic training, working hours/days, customers served/day, and heavy lifting); psychosocial factors (job satisfaction and job stress).

Apart from the main researchers, the study team consisted of four health and safety professional data collectors and one experienced field supervisor. During data collection, the respondents were isolated from their employers and interviews were conducted in a private space. The data collection activities were organized with close follow-up by the principal investigator and supervisor. WMSD data were collected for the preceding 12 months, past one month, and last seven days. Nonetheless, further investigation on the prevalence of WMSDs was only performed for the annual prevalence. In this study, annual prevalence was preferred because it was an appropriate time scale similarly practiced in prior works and was the most commonly used approach as an outcome in other epidemiological MSD studies [6, 13–15, 24].

The quality of data was ensured in various ways. First, forward translation of the Extended Nordic Musculoskeletal Questionnaire was made from English to Amharic by a health professional familiar with the terminology. Questions regarding vehicle repair and maintenance facilities were translated by an automechanical engineer; the approach in the translations emphasized cross-cultural and conceptual translations rather than literal or linguistic equivalence of the terminologies. Afterwards, reverse translation to English language was done by an English language expert to check the consistency. Furthermore, the translated questionnaire and the original English questionnaire were compared and analyzed to identify discrepancies in words, meanings, and contents of the items.

Moreover, a two-day extensive training was given to the supervisor and data collectors on data collection instruments and data collection techniques. In addition to the theoretical session, the training also involved a practical session during which the data collectors visited workshops and rehearsed carrying out some of the activities. The rehearsal sites were vehicle repair workshops outside of the study area in Shashamane town.

The questionnaire was then pretested on 5% of the total sample size outside of the study area in similar workshops in Shashamane town. The main purpose of the pretest was to identify any problems regarding the design and readability of the tool. A secondary objective of the pretest was to ensure that the instrument was interpretable by individuals with or without anatomical knowledge. After the pretest and essential modifications, the tool was finalized. The data from the pretest were not included in the main study.

All the data collected using questionnaires were error checked and coded before they was entered in to the computer database. The data were entered using a data entry template created by using the Epi Info version 7 software. Next, the data were exported to the statistical package for the social sciences software (SPSS) version 20 for cleaning and analysis. Data were edited and cleaned by running a simple frequency, cross tabulations and sorting to check for inconsistencies and completeness and to identify outliers.

For the first specific objective, frequency distribution in number and percentage was used to describe the data. Descriptive findings were presented by frequency tables, graphs, percentage, and proportion with 95% confidence interval (CI).

For the second specific objective to determine the independent factors associated with work-related musculoskeletal disorders, bivariate logistic regression and chi-square test were used to explore presence of statistical association between different independent variables and the outcome variable using crude odds ratio with 95% CI. A *P* value of <0.25 was used as a cutoff point to select the candidate variables for multivariable analysis. The cutoff point was selected to reduce an excessive number of variables and an unstable estimate in the multivariate logistic analyses [25]. Variables with a *P* value of less than 0.05 were considered as statistically significant and presented by the adjusted odds ratio (AOR) with 95% CI in the multivariate analysis.

Collinearity test was checked by running a collinearity diagnostics. Collinearity of each variable in this study was less than 5. This indicates that a specified independent variable was not explained by another independent variable in the model [26]. Model fitting was checked using Hosmer–Lemeshow goodness of test which showed chi-squared test (*X*^2^ = 2.208) with a degree of freedom of 8 and a significance equal to 0.974. The Hosmer–Lemeshow test should be insignificant at *P* value at 0.05 indicating that the variable entered fits the model [27].

Ethical clearance was obtained from the Institutional Review Board (IRB) of Hawassa University College of Medicine and Health Sciences. Before data collection, a permission letter was obtained from the Hawassa city Administration Road and Transport Bureau, Southern Nations, Nationalities, and Peoples' Regional State Bureau of Transport and Road Development and the Federal Road and Transport Authority Liaison Office. In addition, participation of respondents was based on full acceptance and volunteerism, and they were free to decline or withdraw at any time. Finally, maximum efforts were made to keep the privacy and confidentiality of participants at the time of data collection and during analysis.

## 3. Operational Definitions

### 3.1. Vehicle

It includes self-propelled machinery including cars, buses, off-road vehicles, light trucks, and regular trucksthat do not operate on rails (such as trains or trams) which are used for the transportation of people or cargo [28].

### 3.2. Vehicle Repair Worker

The workers were female or male workers, who directly engaged in services that keep vehicle features and systems running smoothly.

### 3.3. Work-Related Musculoskeletal Disorders (WMSD)

WMSD is a self-reported pain, ache, or discomfort for at least 2-3 work days during the past week, past month, or the last 12 months in any part of the neck, shoulder, upper back, lower back, hip/thigh, knee/leg, and ankle/foot and wrist/hand. These symptoms appear at work and often disappear during rest. They may continue after work ends [19]. Disorders caused by slips, falls, motor vehicle accidents, or similar incidents were not considered as WMSD.

### 3.4. Awkward Postures (AP)

AP include working with the neck bent more than 30 degrees without support, working with a bent wrist, working with the back bent without support, and squatting and kneeling for two or more hours [24].

### 3.5. Static Postures (SP)

SP include sitting or standing in a restricted space for two or more hours without changing positions [13].

### 3.6. Job Satisfaction

It is a score measured using the job satisfaction scale as YES (32–45) and NO (10–31) [22].

### 3.7. Job Stress

It is a score measured using the work place stress scale as YES (16 to 40) and NO (lower than or equal 15) [23].

### 3.8. Body Mass Index

Body mass index is calculated as weight in kilograms divided by the square of the height in meters (kg/m^2^):

  Underweight = BMI <18.50
  Normal range = BMI b/n 18.50–24.99
  Overweight = BMI b/n 25.00–29.99
  Obese = BMI = 30.00

### 3.9. Cigarette Smoking

It is a practice of smoking cigarette, at least one stick of cigarette per day [13].

### 3.10. Alcohol Drinking

It is defined as consumption of any kind of alcohol at least for two times per week for different purpose [6].

### 3.11. Khat Chewing

#### 3.11.1. Nonuser

A person who has never used khat in any form.

#### 3.11.2. Current User

A person who was chewing khat within 30 days preceding the study.

### 3.12. Physical Exercise

Physical exercise includes exercise in any kinds of sport activity at least two times per week with a duration of 30 minutes [6].

### 3.13. Repetitive Work

It includes work-related tasks which repeat itself every 30 seconds in the same direction [24].

### 3.14. Assistive Equipment

It includes devices that make the jobs of vehicle repair workers easier, faster, and more efficient (e.g., hydraulics lifts, Jacks stands to support the vehicle once it is elevated, battery chargers, engine hoist, transmission jacks, and pneumatic gun to open nut bolts).

## 4. Results

### 4.1. Sociodemographic Characteristics

A total of 344 vehicle repair workers participated in this study giving a response rate of 99.1%. Of the 344, 340 (98.8%) were male. The mean age of the respondents was 32.7 with standard deviation (SD) ± 8.8 and range between18 and 73. With respect to education, 119 (34.6%) of the respondents had completed their secondary education while 11(3.2%) of them did not attain any formal education. A majority, 181 (52.6%) of the respondents had had between 5 and 15 years of experience in the vehicle repair industry. Concerning monthly income, 95 (27.6%) of the workers had a monthly income of less than 2500 Ethiopian birr (ETH. BR) and 20 (5.8%) had a monthly income above 7500 ETH. BR, with 3200 ETH. BR being the median income (Table 1).

### 4.2. Personal Characteristics of the Respondents

A total of 260 (75.6%) of the participants had a normal BMI, ranging from 18.5 to 24.9 kg/m^2^ while 10 (2.9%) were obese (greater than 30 kg/m^2^). Though a majority of the respondents, 195 (56.7%) and 176 (51.2%), had a drinking and khat chewing habit, respectively, only 117 (34%) were smokers. Regarding physical exercise, 147 (42.7%) of the participants exercised regularly. Respondents were also asked about having a medical history of systemic illnesses. In response, 108 (31.4%) admitted having a medical history of systemic illness (Table 2).

### 4.3. Work Environment and Occupation-Related Characteristics of Respondents

Respondents were asked about the number of hours they spent at the work site per week. A total of 132 (38.4%) reported working more than 48 hours per week while the remaining worked 48 hours or less. Regarding their job category, it was found that 150 (43.6%) of the respondents worked as a mechanical repair worker. Among the respondents, 246 (71.5%) reported as serving less than five customers per day, with four being the median number of customers served. Similarly, slightly over half of the respondents, i.e., (53.5%), stood at least 1–3 hours per day at the work site (Table 3).

### 4.4. Ergonomic and Psychosocial Characteristics of Respondents

**Figure 1:**
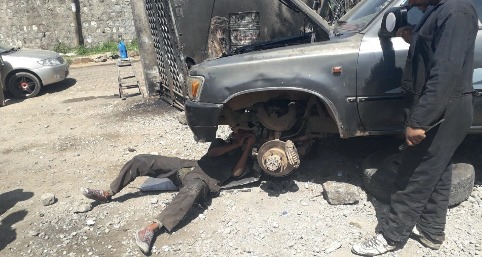
A vehicle repair worker in an awkward posture while working on a vehicle.

**Figure 2:**
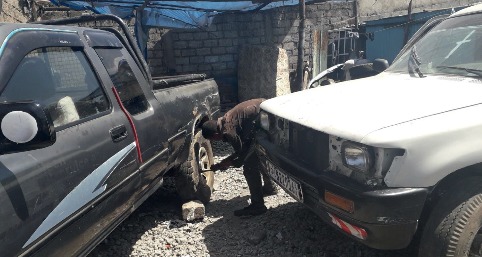
A vehicle repair worker exerting high force to manually tighten the wheels of a vehicle.

**Figure 3:**
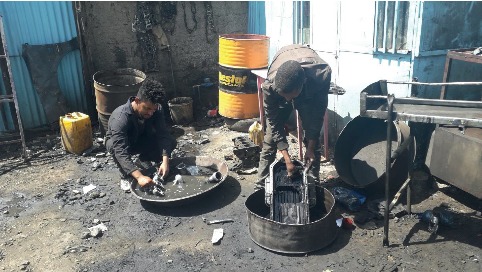
Vehicle repair workers bending and squatting to clean parts.

Regarding working posture, 167 (48.5%) of the respondents worked in the same position for greater than 2 hours per day and 166 (48.3%) of the respondents' job involved always bending/twisting in an awkward way (see Figures 1–3). However, 147 (42.7%) of the participants regularly pushed, pulled, lifted, and moved loads of greater than 20 kg without any one's help or assistive equipment in their daily work. This figure was much higher 262 (76.2%) for loads of greater than 5 kg. Nearly three-fourths (71.5%) of the participants had never received any training with respect to ergonomic postures and safe handling of equipment.

**Figure 4:**
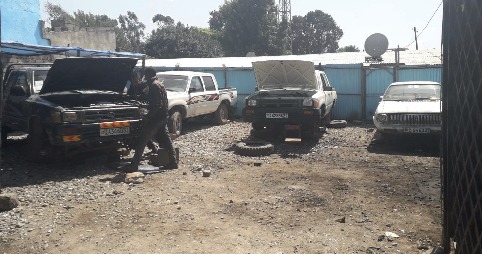
A vehicle repair worker standing while working on a vehicle.

Out of all the participants, 223 (64.8%) reported using assistive equipment during work, while 211 (61.3%) said they exerted force when they used tools and equipment in their work (see Figure 2), and (56.1%) respondents stated as having insufficient space to do their work properly and comfortably. With regard to the most commonly adopted work posture, 128 (37.2%) of the participants favored standing (see Figure 4) while bending and squatting accounted for 103 (29.7%) and 18 (5.8%), respectively (see Figure 3).

Concerning psychosocial characteristics, 143 (41.6%) of the respondents suffered from job stress while 156 (45.3%) respondents stated dissatisfaction with their current occupation (Table 4).

### 4.5. Prevalence of Self-Reported Work-Related Musculoskeletal Disorders

Vehicle repair workers who had experienced trouble (ache, pain, and discomfort) in at least one part of their body over the 12 months prior to the study was 164 (47.7%) with 95% CI (42.7, 53.2), whereas the 30 day and 7 day prevalence were 146 (42.4%) and 61 (17.7%), respectively.

Of the self-reported WMSD pain, ache, or discomfort in the 12 month preceding data collection, the three most prevalent complaints were lower back (103 workers, 62.8%), followed by shoulder complaints (100 workers, 61%) and wrist/hand complaints (53 workers, 32.3%) (Table 5).

Regarding the number of reported disorders, 43 (12.5%) of the vehicle repair workers had WMSDs in one body segment, 37 (10.8%) in two segments, 46 (13.4%) in three segments, 28 (8.1%) in four, and 10 (2.9%) in five segments out of the 9 parts surveyed.

### 4.6. Multiple Body Parts (Right and Left Side) WMSDs

Most of the vehicle repair workers reported pain in multiple body parts (right and left) such as of the shoulder, elbow, hand/wrist, knee, hip/thigh, and feet/ankle. Of the total participants who had shoulder complaints, 40 (24.4%) reported having pain, ache, or discomfort in both shoulders while no respondent reported having elbow pain, ache, or discomfort on both sides of the body (Table 6).

### 4.7. Factors Associated with WMSDs among Vehicle Repair Workers

Each variable was analyzed using bivariate logistic regression, and variables with a *P* value of less than 0.25 were fitted to the multivariable logistic regression. In this regard, eight variables that were eligible for further analysis were entered for multivariate binary logistic regression.

The multivariate binary logistic regression analysis identified professional training, moving loads that weigh greater than 20 kg, repetitive motions, force exertion, and job stress as having significant association with WMSDs. Vehicle repair workers who did not have any professional training were 2.04 times more likely to develop WMSDs than those who had professional training (AOR: 2.04, 95% CI (1.09–3.81)).

The odds of WMSDs among workers who regularly engaged in lifting, pushing, and pulling loads greater than 20 kg without another person's help or assistive tools were 4.85 times greater than that of those who did not regularly engage themselves in the activities (AOR: 4.85, 95% CI (2.65–8.87)). Vehicle repair workers whose tasks always involved repetitive motions were 4.49 times more likely to develop WMSDs than those whose tasks did not involve repetitive motions (AOR: 4.19, 95% CI (1.94–10.4)). Similarly, those who sometimes engaged in repetitive motions were 4.29 times more likely to develop WMSDs compared to vehicle repair workers who never engaged in repetitive motions (AOR: 4.29, 95% CI (1.78–10.2)).

The odds of WMSDs among respondents that exerted excess force while using their tools were 2.4 times more than those that did not. Job stress was also found to be significantly associated with WMSDs, and vehicle repair workers who were stressed because of their job were 4.5 times more likely to develop WMSDs (AOR: 4.54, 95% CI (2.44–8.46)) (Table 7).

## 5. Discussion

### 5.1. Prevalence of Work-Related Musculoskeletal Disorders

In this study, the overall prevalence of self-reported WMSDs among vehicle repair workers was (47.7%) with 95% CI (42.7, 53.2). Not having professional training, moving loads of greater than 20 kg, repetitive tasks, force exertion, and job stress were significantly associated with the prevalence of WMSDs.

The 47.7% annual prevalence of WMSDs obtained in this study was lower than the annual prevalence of WMSD reported in studies conducted in Malaysia (87.4%), India (58%), and Bangladesh (77%) [13, 15, 16]. A comparison of this finding with the findings of other surveys in musculoskeletal epidemiology should take into account the differences in epidemiologic case definitions that may exist among the different studies. Variations in epidemiological case definitions have major impacts on prevalence of common musculoskeletal disorders [29].

Vehicle repair workers reported WMSDs in one or more body segments, with a maximum complaint of the affliction of five body parts. This finding is similar with a finding of a Malaysian study in which complaints about inflictions of the same number of body parts was reported [15]. The presence of multiple disorders is likely caused or exacerbated by the fact that most workers in the developing world use rudimentary appliances that involve manual handling when they maintain, install, dismantle, or even repair heavy materials. All these activities make workers fall victim to risks of MSDs [16].

The most reported pain or discomfort in this study was lower back pain at 62.8%. This was consistent with studies done in Norway, Bangladesh, and Malaysia [15, 16, 24]. The possible explanation for the similarity of the findings could be that often time workers might maintain twisted, bent, and/or other non-neutral trunk postures while working under, inside, and at the sides of a vehicle [24]. Additionally, there is an association between low back pain and working in twisted, bent, and/or other non-neutral trunk postures [6].

Shoulder pain was reported as the second most troublesome disorder at work accounting for 61% of the total complaints. This finding was higher than the 32% prevalence reported in Malaysia [15]. In many repair workshops, it is common among workers to often work with their arms at or above the shoulder level while they are working under the hood [13]. There are also tasks performed underneath the car requiring workers to operate with their arms flexed at or above the shoulder level for long hours [12]. Therefore, it seems reasonable to assume that shoulder symptoms may be caused or aggravated by the physical working environment in garages.

With regards to factors associated with WMSDs, the results of this study indicated that not having professional training is significantly associated with the occurrence of WMSDs. Vehicle repair workers who did not have any professional training were around two times more likely to develop WMSDs than those who had professional training. Clearly, workers who have received professional training are more likely to observe recommended safety rules and to have better awareness about prevention of work-related injuries and disorders [30].

Another important determinant of WMSDs in this study was heavy manual handling. The odds of WMSDs among vehicle repair workers who were regularly engaged in lifting, pushing, and pulling loads greater than 20 kg without another person's help or assistive tools were more than four times than those who did not engage in such activities. This is supported by findings from Bangladesh and Malaysia [15, 16]. This might be explained by the reality in many developing nations where most workers in these areas resort to manual material handling as there is limited access to weight lifting equipment [6]. Studies have reported that workers performing manual material handling are more likely to have musculoskeletal complaints as compared to their counterparts who did not conduct such activities [31].

Similarly, in this study, workers whose tasks always involved repetitive motions were 4.49 times more likely to develop WMSDs. On the contrary, workers who sometimes engaged in repetitive motions more than four times were more likely to develop WMSDs compared to those who never engaged in repetitive motions. Studies have shown that workers performing highly repetitive tasks are at the highest risk for developing of MSDs [6, 13]. High frequency of tasks is a significant risk factor for WMSDs because the worker cannot fully recover in the short periods of time that are given between tasks [32].

Force exertion was one of the ergonomic factors significantly associated with the occurrence of work-related musculoskeletal disorders in the present study. Workers that had a higher level of perceived physical exertion while performing vehicle maintenance operations and operating tools in the work place had more than two times the odds to develop WMSD compared to those who did not. Consistent with this finding, studies conducted in Bangladesh and Malaysia reported that force exertion was a significant predictor in MSD cases [15, 16]. The reason might be because of forceful exertions which require an application of considerable contraction by the muscles, nerves, and tendons which cause them to fatigue rapidly [32].

In the present study, workers who were stressed as a result of their job had more than four times the odds of musculoskeletal disorders in their work place than those with no job-related stress. This is supported by findings from a study conducted in Malaysia that psychosocial factors were significant findings in all types of MSDs [15]. It has previously been reported that the nature of vehicles repair work, poor working conditions, the lack of appropriate safety equipment, and lack of safety training for protection might be stressful for workers [12].

Collecting data from all the eligible vehicle repair workers and the utilization of internationally accepted or validated measurement tools to assess musculoskeletal complaints, job stress, and job satisfaction of the workers were key strengths of this study. Nevertheless, this study is not without its limitations. There is a likelihood of over, under, or misreporting of musculoskeletal complaints in self-reported studies. Furthermore, participant responses may be biased as a result of social desirability to provide sociably preferred answers more than the answers that reflect their real experiences. However, efforts were made to reduce social desirability through ensuring only study participants were present to maintain privacy during the time of data collection. The potential of recall bias related to the time elapsed between the event and data collection time should also be considered even though respondents were allowed as much time as they needed for an adequate recall of long-term memory. For future studies, it is very important to undertake a longitudinal study to produce more scientific evidence that accounts for latency effect, natural history, and prognosis for WMSDs among vehicle repair workers.

Generally, this study showed a 47.7% prevalence of work-related musculoskeletal disorders among vehicle repair workers in the Hawassa city. It is seen that force exertion when using tools, tasks undertaken repetitively, manual handling of loads greater than 20 kg, stress caused by the job, and lack of professional training were the main factors for the existing health problems. Vehicle repair owners and managers of repair establishment should investigate methods to reduce or eliminate risk factors leading to the musculoskeletal disorders found among these workers. Those individuals should also strive to create a good work environment for workers by satisfying their needs as well as educating workers on ergonomics, working body posture, and proper use of tools and equipment. In addition, the workers themselves should practice self-stretching physical exercises during their break time to reduce muscle fatigue and should use lifting devices or helping partners to lift heavy objects.

## Figures and Tables

**Table 1:** Sociodemographic characteristics of vehicle repair workers in Hawassa city, Ethiopia, May 2019.

Categories for variables	Frequency (*n* = 344)	Percentage
Sex		
Male	340	98.8
Female	4	1.2

Age		
18–20	13	3.8
21–29	142	41.3
30–39	117	34.0
40–49	53	15.4
≥50	19	5.5

Educational status		
No formal education	11	3.2
Primary education	104	30.2
Secondary education	119	34.6
Tertiary and above#	110	32.0

Service year		
<5 years	106	30.8
5–15 years	181	52.6
≥16 years	57	16.6

Monthly net income		
<2500 ETH. BR	95	27.6
2500–5000 ETH. BR	196	57.0
5001–7500 ETH. BR	33	9.6
>7500 ETH. BR	20	5.8

Work title		
Senior mechanic	201	70.1
Assistant/apprentice	103	29.9

^#^Tertiary and above include diploma, vocational training, degree, masters, etc.

**Table 2:** Personal characteristics of vehicle repair workers in Hawassa city, Ethiopia, May 2019.

Categories for variables	Frequency (*n* = 344)	Percent
BMI		
Under weight	24	7
Normal weight	260	75.6
Overweight	50	14.5
Obese	10	2.9

Cigarette smoking		
Yes	117	34
No	227	66

Alcohol drinking		
Yes	195	56.7
No	149	43.3

Regular physical exercise		
Yes	147	42.7
No	197	57.3

Practice of khat chewing		
Nonuser	168	48.8
Current user	176	51.2

Professional mechanic training		
Yes	156	45.3
No	188	54.7

Medical history of systemic illness		
Yes	108	31.4
No	236	68.6

**Table 3:** Work environment characteristics of vehicle repair workers in Hawassa city, Ethiopia, May 2019.

Categories of variables	Frequency (*n* = 344)	Percent (%)
Working hours per week		
≤48 hours	212	61.6
>48 hours	132	38.4

Job category/responsibility		
Mechanical repair	150	43.6
Electrical repair	71	20.6
Body beating/wielding	77	22.4
Spray painting	32	9.3
Repair-associated activities^#^	14	4.1

Hours spent standing at work/day		
1–3 hours	184	53.5
4–6 hours	142	41.3
>6 hours	18	5.2

Type of floor at work site		
Concrete	130	37.8
Gravel	125	36.3
No ground cover	89	25.9

Number of customers/day		
<5 customers	246	71.5
≥5 customers	98	28.5

Type of vehicle serviced		
Sedan/saloon cars	59	17.2
Pickups/light trucks	80	23.3
Trucks/large trucks	71	20.6
Bus	44	12.8
Minibus/minivan	47	13.7
SUVs'/4WDs	43	12.5

^#^Repair-associated activities (seat repair and radiator repair work).

**Table 4:** Ergonomic and psychosocial characteristics of vehicle repair workers in Hawassa city, Ethiopia, May 2019.

Categories for variables	Frequency (*n* = 344)	Percent
Bending/twisting in an awkward way		
Never	48	14
Sometimes	130	37.8
Always	166	48.3

Working in the same position for >2 hrs		
Never	48	14
Sometimes	129	37.5
Always	167	48.5

Repetitive motions		
Never	71	20.6
Sometimes	150	43.6
Always	123	35.8

Lift, push, pull, carry, move >5 kg		
Yes	262	76.2
No	82	23.8

Lift, push, pull, carry, move >20 kg		
Yes	147	42.7
No	197	57.3

Training on ergonomics-related issues		
Yes	98	28.5
No	246	71.5

Use of assistive tools		
Yes	235	68.3
No	109	31.7

Exert force while using tools		
Yes	211	61.3
No	133	38.7

Insufficient space to work comfortably		
Yes	193	56.1
No	151	43.9

Most commonly adopted work posture		
Sitting	15	4.1
Standing	128	37.2
Kneeling	49	14.2
Bending	103	29.7
Squatting	18	5.8
Lying on the ground	31	9

Job stress		
Yes	143	41.6
No	201	58.4

Job satisfaction		
Yes	188	54.7
No	156	45.3

**Table 5:** Prevalence of WMSDs in different body segments of vehicle repair workers in Hawassa city, Ethiopia, May 2019 (*n* = 164).

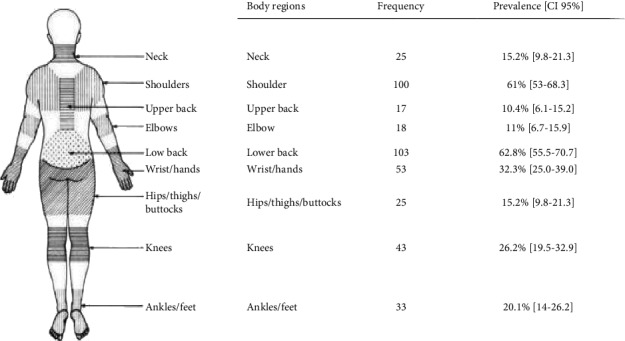

Frequency and prevalence exceed 164 and 100% because of some workers had more than one symptom.

**Table 6:** Multiple body parts (right and left side) WMSDs among vehicle repair workers in Hawassa city, Ethiopia, May 2019.

Body parts	Frequency	Percent (%)
Shoulder		
Both	40	24.4
Left	14	8.5
Right	46	28
No	64	39

Elbow		
Both	0	0
Left	6	3.7
Right	12	7.3
No	146	89

Wrist/hands		
Both	10	6.1
Left	8	4.9
Right	35	21.3
No	111	67.7

Hip/thigh		
Both	10	6.1
Left	11	6.7
Right	4	2.4
No	139	84.8

Knee		
Both	23	14.0
Left	6	3.7
Right	14	8.5
No	121	73.8

Ankle/feet		
Both	21	12.6
Left	7	4.3
Right	5	3
No	131	79.9

**Table 7:** Multivariate logistic regression of factors associated with WMSDs among vehicle repair workers in Hawassa city, Ethiopia, May 2019.

Variable	Work-related MSD		COR (95% CI)	AOR (95% CI)	*P* value
	Yes	No			
Educational status					
No formal education	7 (63.6%)	4 (36.4%)	2.27 (0.75–9.87)	1.07 (0.20–5.51)	0.933
Elementary level	59 (56.7%)	45 (43.3%)	2.04 (1.18–3.52)	1.64 (0.74–3.62)	0.217
Secondary level	55 (46.2%)	64 (53.8%)	1.33 (0.79–2.26)	0.83 (0.38–1.79)	0.648
Tertiary and above	43 (39.1%)	67 (60.9%)	1	1	

Professional training					
Yes	59 (37.8%)	97 (62.2%)	1	1	
No	105 (55.9%)	83 (44.1%)	2.08 (1.34–3.20)	2.04 (1.09–3.81)^*∗*^	0.026

Type of car repaired					
Sedan/saloon car	23 (39.0%)	36 (61.0%)	1	1	
Pickup/light truck	36 (45.0%)	44 (55.0%)	1.28 (0.64–2.53)	0.77 (0.28–2.10)	0.61
Truck/large truck	41 (57.7%)	30 (42.3%)	2.13 (1.05–4.32)	1.02 (0.36–2.92)	0.96
Bus	26 (59.1%)	18 (40.9%)	2.26 (1.01–5.01)	0.87 (0.27–2.77)	0.817
Minibus/minivan	22 (46.8%)	25 (53.2%)	1.37 (0.63–2.99)	1.14 (0.36–3.64)	0.816
SUV/4WD	16 (37.2%)	27 (62.8%)	0.92 (0.41–2.08)	0.75 (0.24–2.33)	0.62

Most commonly adopted posture					
Sitting	5 (35.7%)	9 (64.3%)	1	1	
Standing	57 (44.5%)	71 (55.5%)	1.44 (0.45–4.55)	0.50 (0.12–2.02)	0.344
Kneeling	23 (46.9%)	26 (53.1%)	1.59 (0.46–5.44)	0.973 (0.20–4.66)	0.973
Bending	47 (46.1%)	55 (53.9%)	1.53 (0.48–4.90)	0.921(0.226–3.75)	0.909
Squatting	10 (50.0%)	10 (50.0%)	1.80 (0.44–7.30)	1.17 (0.19–6.89)	0.862
Laying on the ground	22 (71.0%)	9 (29.0%)	4.40 (1.15–16.8)	3.68 (0.70–19.16)	0.121

Lift, push, pull loads of >20 kg					
Yes	106 (72.1%)	41 (27.9%)	6.19 (3.86–9.94)	4.85 (2.65–8.87)^*∗∗∗*^	0.000
No	58 (29.4%)	139 (70.6%)	1	1	

Repetitive motions					
Never	24 (33.8%)	47 (66.2%)	1	1	
Sometimes	71 (47.3%)	79 (52.7%)	1.76 (0.97–3.16)	4.49 (1.94–10.4)^*∗∗∗*^	0.000
Always	69 (56.1%)	54 (43.9%)	2.50 (1.36–4.59)	4.29 (1.78–10.2)^*∗∗∗*^	0.001

Force exertion when using tools					
Yes	122 (57.8%)	89 (42.2%)	2.97 (1.88–4.68)	2.40 (1.24–4.62)^*∗∗*^	0.009
No	42 (31.6%)	91 (68.4%)	1	1	

Job stress					
Yes	93 (65.0%)	50 (35.0%)	3.40 (2.17–5.33)	4.54 (2.44–8.46)^*∗∗∗*^	0.000
No	71 (35.3%)	130 (64.7%)	1	1	

^*∗*^Significant association; significant at ^*∗*^*P* ≤ 0.05, ^*∗∗*^*P* ≤ 0.01, and ^*∗∗∗*^*P* ≤ 0.001.

## Data Availability

The data used to support the findings of this study are available from the corresponding author upon request.

## Abbreviations

BMI: Body mass index
AOR: Adjusted odds ratio
ILO: International Labor Organization
MSD: Musculoskeletal disorders
SNNPR: Southern Nations, Nationalities, and Peoples' Region
WMSDs: Work-related musculoskeletal disorders.
